# Type II Crigler-Najjar syndrome: a case report and literature review

**DOI:** 10.3389/fmed.2024.1354514

**Published:** 2024-05-09

**Authors:** Tao He, Xiaoling Geng, Lei Zhu, Xue Lin, Lixia Wang

**Affiliations:** Department of Gastroenterology, The First Affiliated Hospital of Dalian Medical University, Dalian, China

**Keywords:** Crigler-Najjar syndrome, hyperbilirubinemia, UGT1A1, phenobarbital, case report

## Abstract

**Background:**

Crigler-Najjar syndrome (CNS) is caused by mutations in uridine 5′-diphosphate glucuronyltransferase (UGT1A1) resulting in enzyme deficiency and hyperbilirubinemia. Type II CNS patients could respond to phenobarbital treatment and survive. This study presents a rare case of type II CNS.

**Case summary:**

The proband was a 29-year-old male patient admitted with severe jaundice. A hepatic biopsy showed bullous steatosis of the peri-central veins of the hepatic lobule, sediment of bile pigment, and mild periportal inflammation with normal liver plate structure. The type II CNS was diagnosed by routine genomic sequencing which found that the proband with the Gry71Arg/Tyr486Asp compound heterozygous mutations in the UGT1A1 gene. After treatment with phenobarbital (180 mg/day), his bilirubin levels fluctuated between 100 and 200 μmol/L for 6 months and without severe icterus.

**Conclusion:**

Type II CNS could be diagnosed by routine gene sequencing and treated by phenobarbital.

## Introduction

Crigler-Najjar syndrome (CNS) is an inherited deficiency of uridine 5′-diphosphate glucuronyltransferase (UGT1A1) enzyme and is characterized by hyperbilirubinemia. The complete absence of the UGT1A1 enzyme activity results in type I, which does not respond to phenobarbital and was reported in 1952 by Crigler and Najjar, while a partial loss of UGT1A1 activity results in type II, first found by Arias and that can respond to phenobarbital treatment (1, 2). The major manifestation of CNS is jaundice. Type I CNS patients usually die in infancy, but type II CNS patients survive despite long-term icterus and pruritus. This study presents a rare case of type II CNS.

## Case presentation

**Figure 1:**
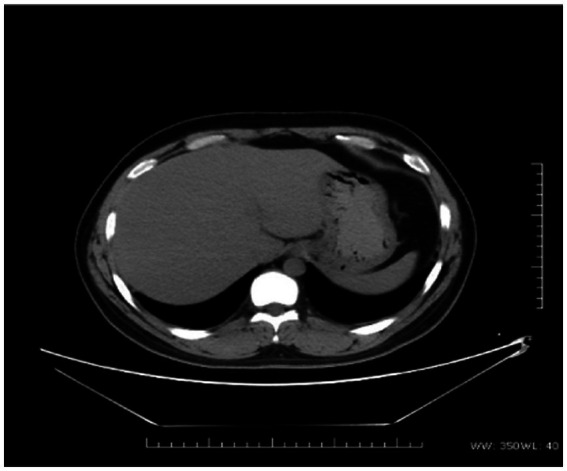
The CT scan of the proband. Lower density and normal shape of the liver (CT index is 50 U).

**Figure 2:**
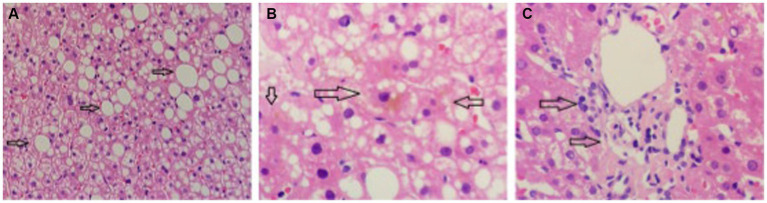
The pathology of liver biopsy. **(A)** Normal hepatic plate structure with bullous steatosis of peri-central veins of hepatic lobule (indicated by arrow). **(B)** Sediment of bile pigment (indicated by arrow). **(C)** Mild periportal inflammation.

The proband was a 29-year-old male patient admitted to First Affiliated hospital of Dalian Medical University with severe jaundice. His parents claimed that he had icterus and pruritus from birth, without any treatment. He was 163 cm in height and 81 kg in weight. All hemolytic anemia parameters, including Hemoglobin, coagulation function, thyroid function, ceruloplasmin, and immunoglobulin was normal. Total bilirubin was 237.8 μmol/L, among which unconjugated bilirubin was 228.8 μmol/L, other parameters of liver function were all in normal range. Hepatitis, Epstein–Barr virus, and cytomegalovirus tests were negative. Computed tomography (CT) scan showed a low density and normal shape of the liver. The CT index was 50 HU, suggesting fatty liver (Figure 1). A hepatic biopsy showed bullous steatosis of the peri-central veins of the hepatic lobule, bile pigment sedimentation, and mild periportal inflammation with normal liver plate structure (Figure 2).

There is no consanguinity between the proband’s parents. To confirm the diagnosis of type II CNS, blood genomic DNA from the proband and his parents was routinely extracted and sequenced. The genetic test results showed two suspected homozygous pathogenic mutations. One mutation was the c.1456 T > G p.Y486D homozygous mutation. Y486D is located on exon 5, changing the 1,456 thymine (T) into guanine (G) and changing residue 486 tyrosine (Tyr) into aspartic acid (Asp). His parents were c.1456 T > G p.Y486D heterozygous carriers (Supplementary Figures S1A–C). The other mutation was the c.211G > A p.G71R homozygous mutation. G71R is located in exon 1, mutating the 211 guanine (G) into adenine (A) and changing residue 71 from glycine (Gly) to arginine (Arg). His father was a c.211G > A p.G71R homozygous carrier without any symptoms, and his mother was a heterozygosis carrier (Supplementary Figures S1D–F).

The patient was therefore diagnosed with type II CNS. He received phenobarbital treatment (180 mg/day) since then to reduce bilirubin levels associated with jaundice. After that, his mean total bilirubin levels fluctuated between 100 and 200 μmol/L during the next 6 months and without severe icterus and pruritus without severe side effects.

## Discussion

This study presents a rare case of type II CNS. Hepatic biopsy showed bullous steatosis of the peri-central veins of the hepatic lobule, sediment of bile pigment, and mild periportal inflammation with normal liver plate structure. The type II CNS was diagnosed by routine genomic sequencing which found that the proband with the Gry71Arg/Tyr486Asp compound heterozygous mutations in the UGT1A1 gene. After treatment with phenobarbital, the bilirubin levels fluctuated between 100 and 200 μmol/L for 6 months and without severe icterus.

The molecular basis of type II CNS is mainly missense *UGT1A1* gene mutations resulting in the partial lack of UGT enzyme activity (3). Many single-nucleotide mutations are responsible for type II CNS in East Asians, particularly in the first exon with the Gry71Arg mutation. The carrier rate in the East Asian people is as high as 15.2% and even 30% in the Chinese people (4). Multiple mutations in different areas of the *UGT1A1* gene and compound heterozygous mutations can cause type II CNS (5). Yamamoto et al. reported that different combinations of the Gry71Arg and Tyr486Asp mutations could result in different enzymatic activities. The UGT activity levels in patients with the Gry71Arg heterozygous, Gry71Arg homozygous, Tyr486Asp homozygous, and Gry71Arg/Tyr486Asp compound heterozygous mutations were reported to be 60.2, 32.2, 7.62%, and 6.2 ± 1.6%, respectively (6). In the case reported here, the patient had the compound heterozygous mutations Gry71Arg + Tyr486Asp. Both Gry71Arg and Try 486Asp were detected in an adult with normal promoter area and continuous jaundice. Therefore, type II NCS could be confirmed. This study summarized the polymorphism markers in the *UGT1A1* gene found in type II CNS (Table 1).

**Table 1:** Reported mutations in UGT1A1 gene in Crigler-Najjar syndrome II.

Author	Date	Country	Mutation	References
Labrune et al.	1994	France	G308E Q357R S381R A401P Q357X W335X+ A368T W335X+ 1223insG A291V K437X	(35)
Kadakol et al.	2000	United States	c. 115C > G c. 222C > A c. 517delC c. 722-723delAG c. 1046delA c. 1223delA/N c. 1451G > A c.1452G > A c. 1,490 T > A/N	(3)
Maruo et al.	2006	Turkey	T-3279G A(TA)7TAA p.H39D	(37)
Huang et al.	2006	Taiwan, China	c.479 T > A c.610A > G c.1465 T > G + c.211G > A	(34)
D’Apolito et al.	2007	Italy	c.835A > T c.1381 T > C c.1328 T > C c.1223–1,224 ins G + c.1184G > T c.1060 T > A	(38)
Sneitz et al.	2010	Finland	c.44 T > G c.1489delG c.1160C > A c.211G4A, c1456T4G, c.1220delA c.625C > T c.572C > T c.1328 T > C c.1006C > A	(39)
Maruo et al.	2011	Turkey	p.K402T + p.G71R;Y486D	(40)
Nakagawa et al.	2011	Japan	c.1456 T > G	(41)
Minucci et al.	2013	Italy,	c.1099C > T+ c.508_510delTTC	(42)
Nilyanimit et al.	2013	Thailand	[A(TA)7TAA]	(43)
Zheng et al.	2014	China	c.211G > A + c.508_510delTTC + c.1456 T > G	(44)
Maruo et al.	2015	Iran	p.V225G	(45)
Tesapirat et al.	2015	Thailand	c.1069-1070insC + c.1456 T > G	(36)
Wu et al.	2016	China	c.610 A > G+ c.1091 C > T	(20)

Both types I and II CNS can occur in children and adults, with clinical jaundice due to elevated total bilirubin, while transaminase can be normal. Due to severe reduction or even complete lack of the UGT, type I CNS can manifest with bilirubin >342 μmol/L and deteriorate with kernicterus and death within 2 years after birth. In type II CNS, jaundice is usually lighter, and total bilirubin is usually <342 μmol/L, but the levels fluctuate because of stress, fatigue, infection, pregnancy, or drug use. Genetic testing helps with the diagnosis. Serum bilirubin levels can decrease by >30% with phenobarbital treatment, with a relatively far better prognosis (20–22). Type II CNS can increase the risk of gallbladder stones. Recently, Fernandes et al. (23) reported that the disease is associated with acute cholangitis. Zhang et al. (24) reported a patient with stenosing papillitis and acute biliary pancreatitis. Regarding the hepatic pathology findings, most cases show small amounts of cystic granules sediment in liver cells under the light microscope, and bile embolism can be found with both electron and light microscope (24). Elfar et al. (25) presented a patient with persistent unconjugated hyperbilirubinemia, clinically diagnosed as type II CNS, who underwent liver transplantation due to liver cirrhosis. They reported that CNS could progress to hepatic fibrosis and cirrhosis (25).

Gilbert’s syndrome is another hereditary condition that affects bilirubin processing by the liver, similar to Crigler-Najjar syndrome, but it is much more common and generally milder. It results from a variation in the same UGT1A1 gene responsible for Crigler-Najjar syndrome, but the impact on bilirubin processing is less severe. Individuals with Gilbert’s syndrome typically have slightly higher bilirubin levels, which may lead to mild jaundice, especially during times of illness, fasting, or stress. However, Gilbert’s syndrome is often considered a benign condition that does not require treatment and does not lead to serious health problems.

The mention of Gilbert’s syndrome is relevant because it lies on the same spectrum of bilirubin metabolism disorders as Crigler-Najjar syndrome, with Gilbert’s at the milder end and Type I Crigler-Najjar at the most severe (26). Type II Crigler-Najjar syndrome falls in between these two. Understanding Gilbert’s syndrome can help clarify the range of genetic variations affecting bilirubin metabolism and their impacts (2).

Regarding consanguinity, this is a relevant consideration for genetic disorders like Crigler-Najjar syndrome. Consanguinity (marriage or reproduction between closely related individuals) increases the likelihood of both parents carrying the same genetic mutation and, therefore, increases the risk of their children inheriting autosomal recessive conditions like Type I Crigler-Najjar syndrome or, to a lesser extent, the genetic variations responsible for Type II Crigler-Najjar syndrome and Gilbert’s syndrome (27). While Gilbert’s syndrome often results from a common variation in the UGT1A1 gene promoter region and might not be directly tied to consanguinity, Type I and Type II Crigler-Najjar syndromes result from more specific mutations that could indeed have a higher incidence in populations or families where consanguinity is more common. Genetic counseling can provide families with information on the risk of inherited conditions and guidance on management and prevention (28).

In a cohort of 22 older patients with type I CNS, nine (41%) were found with histological fibrosis discovered in the explants at the time of liver transplantation. In addition, portal, pericentral, and mixed fibrosis could be seen. Moreover, fibrotic individuals were notably older than those without fibrosis, suggesting that the injury might be incrementally acquired (29).

First and the basic management of CNS is avoiding drugs like penicillin, sulphonamides, salicylates, ceftriaxone, and furosemide that can displace bilirubin from albumin (30). The management of type II CNS is relatively easier and more effective because of the partial activity of the UGT enzyme. Phototherapy and phenobarbital are the most common and effective ones (31). The management of type I CNS is very complicated, and transplantation and gene therapy were only experimental treatments for type I CNS (32). Phototherapy acts by converting bilirubin without the conjugation in the liver. The bilirubin can be excreted in the bile directly. Bilirubin levels over 171 μmol/L are an indication for phototherapy in term infants without risk factors, while the threshold is 4 mg/dL (68.4 μmol/L) in infants with high risk for kernicterus (preterm, low birth weight) (33). This technique involves the patient’s exposure to light for 10–12 h, even 14 h, per day (19, 34, 35). Oral calcium supplementation makes phototherapy more efficient (36). Phenobarbital can induce UGT1A1 activity, and it is the most effective drug for CNS, especially type II CNS. The dose is 3–5 mg/kg/day, usually 60–180 mg/day (5). The dose can be reduced (below 60 mg/day, 30–60 mg/day recommended) in pregnancy to avoid its teratogenic side effects. A response usually can be seen within 2–3 weeks. In addition, calcium supplementation has also been found to increase the gut excretion of bilirubin. Maternal bilirubin serum levels should be below 10 mg/dL (171 μmol/L). Furthermore, folic acid at a dose of 10 mg is recommended during pregnancy (37). Orthotopic liver transplantation (OLT) can offer an option in genetic diseases involving the liver, and many monogenic diseases can be cured by liver transplantation. Kayler et al. (38) firstly retrospectively analyzed the efficiency of OLT in metabolic liver disease, including CNS, and proved beneficial in a 78-month follow-up. Bayram et al. (39) conducted a 24-month research and suggested that OLT should be performed before neurobehavioral abnormalities occur. The mortality is up to 10% in the first post-transplantation year, and lifelong immunosuppressive drugs are needed (2). In a 26-month follow-up, OLD could maintain normal serum bilirubin and no requirement for phototherapy (38). Apart from liver transplantation, liver cell transplantation (LCT) is a promising technique and is less invasive. Chen et al. (39) reported a decline of serum bilirubin by 30–60%, and biliary excretion of bilirubin glucuronides indicated that transplanted iHeps expressed UGT1A1 activity, a postnatal function of hepatocytes. In another research, the efficacy and overall safety of heterologous human adult liver-derived progenitor cell (HepaStem) were confirmed after 12 months (40). A phase I/II prospective, open-label, multicenter, randomized trial aimed primarily at evaluating the safety of HepaStem in pediatric patients with urea cycle disorders (UCDs) or CNS 6 months post-transplantation. The secondary objective included the assessment of safety up to 12 months post-infusion and of preliminary efficacy (41). Table 2 presents the studies of liver transplantation in CNS. Gene therapy can modify the genome, and it is a promising approach to cure gene-related diseases such as CNS. Bellodi-Privato et al. (58) reported a successful gene therapy of the Gunn rat by *in vivo* neonatal hepatic gene transfer using murine oncoretroviral vectors. In the report, the Gunn rats were injected with viruses carrying a functional *UGT1* gene, and bilirubinemia was normal after 6 weeks (3 μmol/L) and remained in the normal range (i.e., <10 μmol/L) for more than 34 weeks (58). Many studies confirmed the efficacy of gene therapy with adeno-associated viruses, including AVV1, AVV2, AVV6, AVV8, VV9, AVV5 (59–61).

**Table 2:** Liver transplantation in the Crigler-Najjar syndrome patient.

Author	Date	Number of cases	Follow up	Result	OLT/LCT*	Reference
Kadakol et al.	2000	1	3 years	Beneficial	OLT	(3)
Schauer et al	2002	2	27 months	Beneficial	OLT	(46)
Al Shurafa et al.	2002	6	1 year	Beneficial and curative	OLT	(47)
Kayler et al.	2002	1	78 months	Beneficial	OLT	(23)
Schauer et al.	2003	3	36 months	Beneficial/Improved	OLT	(48)
Darwish et al.	2004	1	12-month	Beneficial on plasma bilirubin level	LCT	(49)
Ambrosino et al.	2005	1	24 months	Good liver function	LCT	(50)
Broering et al.	2005	2	8 months	Normal total bilirubin and liver function	OLT	(51)
Morioka et al.	2005	2	14 months	Beneficial	OLT	(52)
Strauss et al.	2006	4	20-month	Beneficial	OLT	(20)
Quaglia et al.	2008	1	Not mentioned	Partially Improved	LCT	(53)
Allen et al.	2008	1	26 months	Normal serum bilirubin	OLT	(26)
Khan et al.	2008	1	2 months	Safety, feasibility, and efficacy	LCT	(54)
Ozçay et al.	2009	4	24 months	Curative and beneficial	OLT	(55)
Meyburg et al.	2010	1	15 months	Beneficial	LCT	(56)
Shanmugam et al.	2011	1	18 months	Beneficial	OLT	(57)
Tu et al.	2012	1	12 months	Beneficial	OLT	(58)
Bayram et al.	2013	2	24 months	Successful but irreversible neurobehavioral abnormalities	OLT	(24)
Mauruo et al.	2015	2	—	Death	OLT	(45)
Jorns et al	2015	1	1 year/2 weeks	Good condition with excellent graft function.	LCT	(59)
Baris et al	2018	1	3 months	Beneficial	OLT	(60)
Mitchell et al.	2018	22	5.8 years(mean)	Beneficial	OLT	(13)
Nader et al.	2018	1	6 months	Death of sepsis	OLT	(61)
Elfar et al.	2019	1	Not mentioned	Beneficial	OLT	(12)

*OLT, Orthotopic liver transplantation; LCT, liver cell transplantation.

Gilbert syndrome is an another Missense UGT1A1 gene mutations resulting in the partial lack of UGT enzyme activity.

In conclusion, for type II CNS, phototherapy therapy and drug therapy like phenobarbital are effective, and the prognosis can be satisfying. On the other hand, for type I CNS patients, liver transplantation may be needed eventually. Many new therapeutic approaches are being pursued in preclinical research for developing safe and effective treatments for CNS. Gene therapy has been successfully performed in animals, and the safety and efficacy issues are being identified. Gene therapy might be a promising and realistic modality for the treatment of CNS in the following decades.

## Data availability statement

The original contributions presented in the study are included in the article/Supplementary material, further inquiries can be directed to the corresponding authors.

## Ethics statement

The studies involving humans were approved by the Institutional Review Board of the First Affiliated Hospital of Dalian Medical University. The studies were conducted in accordance with the local legislation and institutional requirements. The participants provided their written informed consent to participate in this study. Written informed consent was obtained from the individual(s) for the publication of any potentially identifiable images or data included in this article.

## Author contributions

TH: Writing – review & editing, Writing – original draft, Supervision, Methodology, Data curation. XG: Writing – review & editing, Writing – original draft, Data curation. LZ: Writing – original draft, Investigation, Conceptualization. XL: Writing – review & editing, Writing – original draft, Methodology. LW: Writing – review & editing, Writing – original draft, Conceptualization.
