# Supplementary material for: Type II Crigler-Najjar syndrome: a case report and literature review
Source: Front Med (Lausanne). 2024 May 9;11:1354514. doi: 10.3389/fmed.2024.1354514 (PMC11112071; doi:10.3389/fmed.2024.1354514)
Supplement: Supplementary file 1 [file Data_Sheet_1.doc]

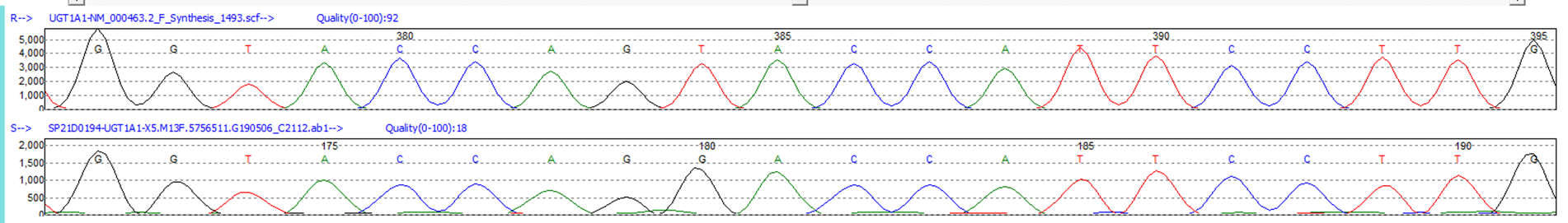
A


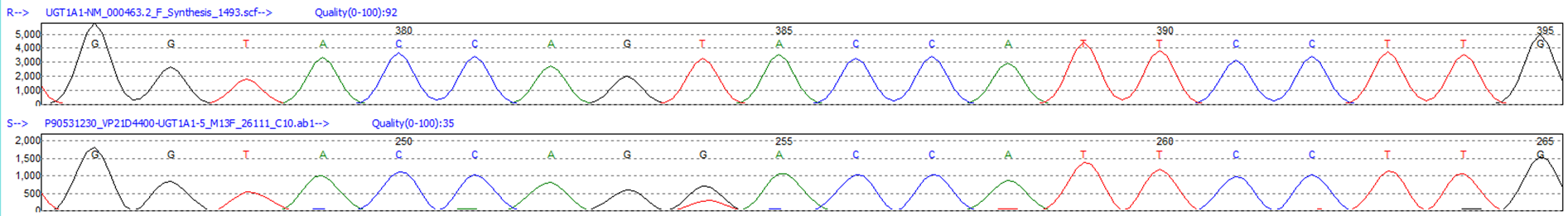
B


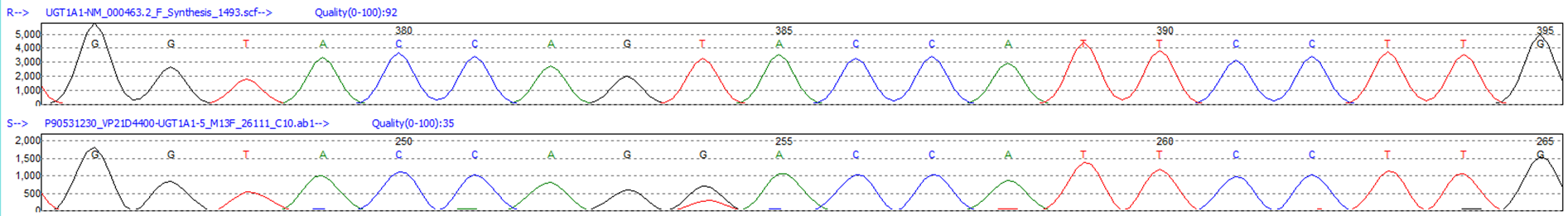


C


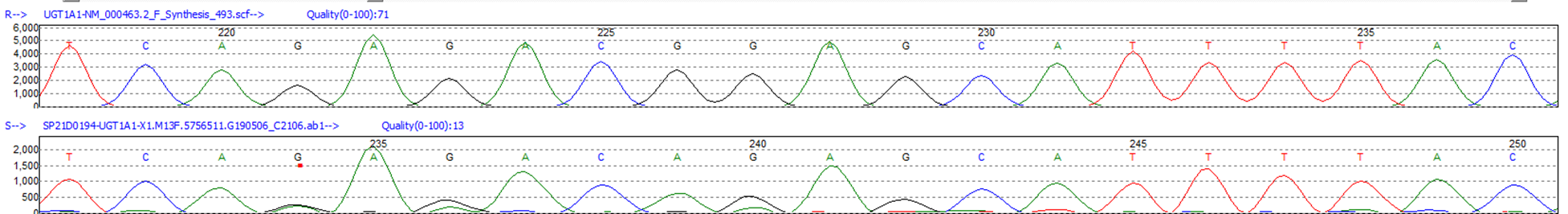


D


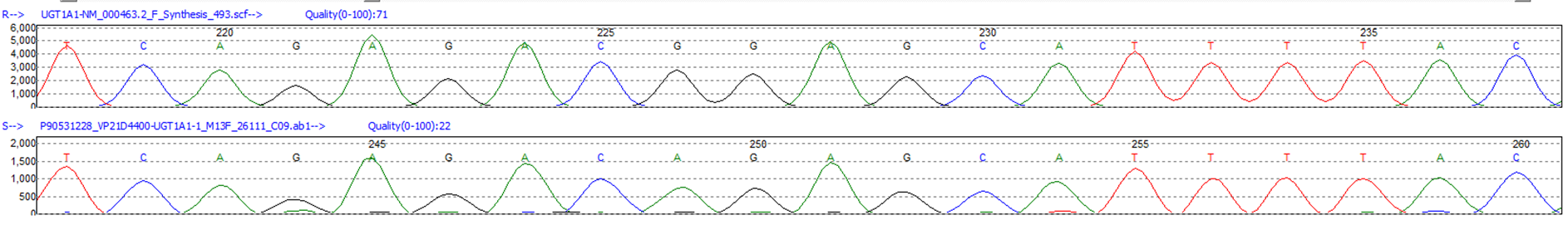


E


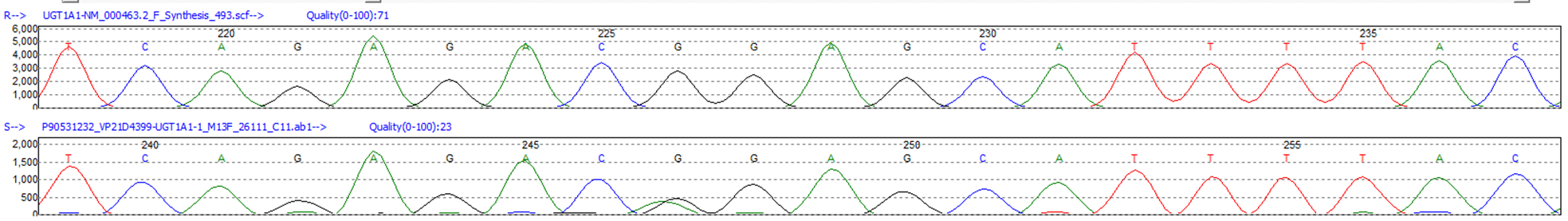


F

**Fig.S1** The gene sequencing of proband and his parents.

(**A**): The gene sequencing of proband (c.1456T>G, p. Y486D). R: Normal sequence; S: Sequence of the proband：No.1456 homozygous mutation is G／G(indicated by arrow above)；

(**B**): The gene sequencing of proband’s father (c.1456T>G, p. Y486D). R: Normal sequence; S: Sequence of proband’s father: No.1456 heterozygosis mutation is T/G (indicated by arrow above)

(**C**): The gene sequencing of proband’s mother (c.1456T>G, p. Y486D), R: Normal sequence, S: Sequence of proband’s mother: No.1456 heterozygosis mutation is T/G (indicated by arrow above).

(**D**): The gene sequencing of proband (c.211G>A, p. G71R), R: Normal sequence, S: Sequence of proband: No. 211 homozygous mutation is G/A (indicated by arrow above)

(**E**): The gene sequencing of proband’s father (c.211G>A, p. G71R), R: Normal sequence, S: Sequence of proband’s father: No. 211 homozygous mutation is G/A (indicated by arrow above)

(**F**): The gene sequencing of proband’s mother (c.211G>A, p. G71R), R: Normal sequence, S: Sequence of proband’s mother: No. 211 heterozygosis mutation (indicated by arrow above
